# Measuring health workers’ motivation composition: validation of a scale based on Self-Determination Theory in Burkina Faso

**DOI:** 10.1186/s12960-017-0208-1

**Published:** 2017-05-22

**Authors:** Julia Lohmann, Aurélia Souares, Justin Tiendrebéogo, Nathalie Houlfort, Paul Jacob Robyn, Serge M. A. Somda, Manuela De Allegri

**Affiliations:** 1 Institute of Public Health, Faculty of Medicine, Heidelberg University, Im Neuenheimer Feld 324, Heidelberg, Germany; 2 Centre de Recherche en Santé de Nouna, BP 02, Nouna, Burkina Faso; 3 Département de Psychologie, Université du Québec à Montréal, C.P. 8888 succursale Centre-ville, Montréal, Québec H3C 3P8 Canada; 4 World Bank, Health, Nutrition, Population Global Practice, 1818H Street, NW Washington, DC 20433 United States of America; 5 Département de Recherche Clinique, Centre MURAZ, 2054 Avenue Mamadou Konaté, 01 BP 390, Bobo-Dioulasso, Burkina Faso

**Keywords:** Health worker motivation, Motivation composition, Measurement, Validation, Self-Determination Theory

## Abstract

**Background:**

Although motivation of health workers in low- and middle-income countries (LMICs) has become a topic of increasing interest by policy makers and researchers in recent years, many aspects are not well understood to date. This is partly due to a lack of appropriate measurement instruments. This article presents evidence on the construct validity of a psychometric scale developed to measure motivation composition, i.e., the extent to which motivation of different origin within and outside of a person contributes to their overall work motivation. It is theoretically grounded in Self-Determination Theory (SDT).

**Methods:**

We conducted a cross-sectional survey of 1142 nurses in 522 government health facilities in 24 districts of Burkina Faso. We assessed the scale’s validity in a confirmatory factor analysis framework, investigating whether the scale measures what it was intended to measure (content, structural, and convergent/discriminant validity) and whether it does so equally well across health worker subgroups (measurement invariance).

**Results:**

Our results show that the scale measures a slightly modified version of the SDT continuum of motivation well. Measurements were overall comparable between subgroups, but results indicate that caution is warranted if a comparison of motivation scores between groups is the focus of analysis.

**Conclusions:**

The scale is a valuable addition to the repository of measurement tools for health worker motivation in LMICs. We expect it to prove useful in the quest for a more comprehensive understanding of motivation as well as of the effects and potential side effects of interventions intended to enhance motivation.

**Electronic supplementary material:**

The online version of this article (doi:10.1186/s12960-017-0208-1) contains supplementary material, which is available to authorized users.

## Abstract French

**Contexte:**

La motivation des agents de santé dans les pays à revenu faible et intermédiaire est devenue un sujet de grand intérêt pour les décideurs et les chercheurs au cours des dernières années. Pourtant, de nombreux aspects de la motivation des agents de santé ne sont pas encore bien compris. Ceci est dû en particulier à l’absence d’outils de mesure appropriés. Cet article présente une preuve de la validité conceptuelle d’une échelle psychométrique développée pour mesurer la composition de la motivation, c’est-à-dire le degré auquel des types de motivation d’origine différente à l’intérieur et à l’extérieur d’une personne contribuent à leur motivation globale au travail. L’échelle est fondée sur la théorie de l’auto-détermination (Self-Determination Theory).

**Méthodes:**

Une enquête transversale a été mise en place auprès de 1 142 infirmières dans 522 formations sanitaires gouvernementales de 24 districts du Burkina Faso. Par analyse factorielle confirmatoire, nous avons examiné si l’échelle mesure ce qu’elle était supposée mesurer (validité structurelle et convergente/discriminante) et si ses propriétés de mesures sont comparables dans différentes sous-groupes d’agents de santé (invariance de la mesure).

**Résultats:**

Les résultats montrent que l’échelle mesure une version légèrement modifiée du continuum de motivation proposée par la théorie de l’auto-détermination. Les propriétés de mesure étaient globalement comparables entre les sous-groupes, mais une certaine prudence est indiquée si une comparaison des moyennes entre les groupes est l’objectif principal de l’analyse.

**Conclusion:**

L’échelle est une un apport important au référentiel des outils de mesure de la motivation des agents de santé dans les pays à revenu faible et modérés. Elle sera utile pour une meilleure compréhension de la motivation des prestataires, ainsi que des effets positifs et potentiellement secondaires des interventions visant à renforcer la motivation.

## Background

Recent years have witnessed an increased awareness of the paramount importance of a motivated health workforce for the functioning of health systems, particularly in countries burdened by severe resource limitations [1]. Interventions targeting health worker motivation such as performance-based financing (PBF) have become extremely popular among policy makers in low- and middle-income countries (LMICs) [2, 3]. Despite the attention such interventions are receiving, gaps in understanding remain. In particular, the mechanisms through which interventions bring about motivational changes and potential side effects thereof remain poorly understood [4–8]. For instance, there is an ongoing debate around whether the monetary incentives involved in PBF undermine intrinsic motivation (“crowding out effect”) [5].

The limited availability of context-adapted research tools to study motivation is a major factor contributing to this knowledge gap. Research on health worker motivation in LMICs has mostly focused on the overall amount or on determinants and outcomes of motivation, leaving other relevant dimensions discussed in the psychological literature such as motivation composition relatively unexplored [4, 9]. Corresponding quantitative measurement tools (e.g., [10–13]), while without doubt useful to answer many research questions, are not suited to others, including that around the crowding out effect which deals with a shift in motivation composition from intrinsic to extrinsic forms.

Against this background, this article contributes to expanding the methodological repository for health worker motivation research by presenting evidence on the construct validity of a newly developed psychometric scale to measure health worker motivation composition. We define motivation composition as the extent to which motivation of different origin within and outside a person contributes to their overall work motivation. The scale is theoretically grounded in Deci and Ryan’s Self-Determination Theory (SDT) [4, 14] and was developed for use in questionnaires or structured interviews. It assesses general motivation towards work rather than task- or situation-specific motivation. The article presents evidence for the scale’s validity from a structured survey with nurses in Burkina Faso. Table 1 contains our specific research questions.

**Table 1:** Aspects of validity investigated and specific research questions

Type of validity	Research questions
Structural validity	RQ1: Is the assumed internal theoretical structure of motivation (i.e., the SDT continuum of motivation; Fig. 1) represented in the data as it was intended during scale development? a. Do respondents distinguish the five dimensions of motivation? b. Are adjacent dimensions more closely related than non-adjacent dimensions?
Generalizability	RQ2: Do psychometric properties and interpretations generalize across health worker subgroups (measurement invariance)?
Convergent and discriminant validity	RQ3: To what extent do relationships between the motivation measure and measures of other related constructs correspond to what is theoretically expected and has been found in previous research with other, established measures?

### The self-determination continuum of motivation

**Fig. 1:**
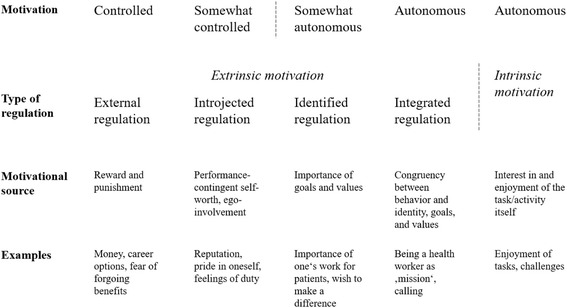
The self-determination continuum of motivation. Legend: adapted from [15, 16]

Self-Determination Theory was introduced in the mid-1980s as a general framework of human motivation [14] and has since been extensively studied and further refined [15]. As part of the overall theory, SDT proposes the self-determination continuum of motivation (Fig. 1), a taxonomy of five major dimensions of motivation that are distinguished by the extent to which they stem from contingencies outside the person (controlled motivation) or originate within the person (autonomous motivation) [16].

The scale validated in this article measures these five motivation dimensions. Motivation originating fully within the person, such as pure enjoyment of a task, is termed intrinsic motivation in SDT. Extrinsic motivation, in contrast, refers to motivation derived from an instrumental purpose of behavior. External regulation corresponds to what is usually referred to as extrinsic motivation: the wish to attain or avoid some consequence. SDT differentiates three additional dimensions of extrinsic motivation by the degree to which the associated contingencies have become part of the person’s self: introjected regulation refers to motivation derived from self-pride, reputation, or feelings of duty, identified regulation to motivation driven by recognition of the importance of one’s job, and integrated regulation to full congruency between one’s personal goals and values and those of one’s job. They differ from external regulation in that they do not need to be maintained from the outside through rewards or punishment. However, they are not fully intrinsic as corresponding behavior is instrumental in catering to a person’s set of values and goals rather than performed out of pure interest or enjoyment. A large body of research has linked autonomous forms of motivation to more favorable performance and other outcomes (e.g., wellbeing, organizational commitment) than controlled forms of motivation [9, 15, 17].

The validity and usefulness of the SDT taxonomy has been confirmed in a wide range of work settings, although mostly in North America and Europe [15]. However, the few studies from LMIC (non-healthcare) settings [18] and the (non-SDT-based) literature on health worker motivation in LMICs suggest its validity in LMIC healthcare contexts as well. Specifically, sources of motivation identified by the latter correspond well to the five dimensions differentiated by the SDT taxonomy (e.g., [4, 7, 8, 10–13, 19–25]). For a theoretical application of SDT and the taxonomy to LMIC healthcare settings, see [4].

## Methods

### Study context

Burkina Faso’s healthcare delivery system relies primarily on the public sector which manages approximately 80% of healthcare facilities. Primary healthcare services are mostly provided by nurses, midwives, and assistant nurses and midwives. Like many other LMICs, Burkina Faso’s health system suffers from multiple challenges including a shortage of certain health worker cadres, their unequal geographical distribution, and challenging working conditions including low pay, substandard infrastructure and equipment, poor supervision, shortages in drugs and other supplies, and few incentives for individual high performance [26–28]. In 2014, the Ministry of Health with support from the World Bank implemented a PBF pilot intervention to strengthen the healthcare system by addressing some of these challenges. Our study took place in the context of the impact evaluation of this intervention.

### Motivation composition measure

The psychometric scale to measure motivation composition was developed by our research team prior to the validation study presented in this article. A detailed description of this process can be found in Additional file 1. A pretest confirmed the scale’s content validity, supporting the validity of the SDT taxonomy in the context and affirming that the items cover the five motivation constructs well and in context-appropriate language.

Similar to other SDT-based measures (e.g., [18, 29]), the scale’s measurement rationale is grounded in the idea that individuals will reveal their underlying motivation composition in the reasons for the actions they provide. Following an introduction, a reflective exercise, and a guiding question (“Why are you motivated to work?”), respondents are thus presented with 26 reasons for which they might be motivated to work (4–12 per motivation dimension; see Additional file 1). They are asked to indicate, on an 11-point scale and with a visual aid, the degree to which each of these reasons are important for their personal work motivation. Respondents’ answers are then used to derive an estimate of their underlying motivation level on the five dimensions.

In order to counteract diverse response biases, we used a hybrid mode of administration, with interviewers reading out instructions and items but interviewees recording their own answers on a separate questionnaire copy. The questionnaire was administered in French in light of the high French proficiency level of Burkinabé health workers. One explicit aim of the validation analyses was the selection of a subsample of items for a final shorter and easy-to-administer scale.

### Sample

We assessed the scale’s validity with data from a structured health worker survey implemented between October 2013 and March 2014 in the context of the abovementioned PBF impact evaluation baseline. The sampling strategy was aligned with the cluster sampling strategy of the impact evaluation accordingly [30]. Data was collected from approximately two thirds of all government health facilities in 24 districts of six regions of the country. Research assistants were instructed to interview all nurses, midwives, and assistant nurses and midwives in 498 primary as well as selected staff in 24 secondary-level facilities present on the day of the study team visit. Fifty-five per cent of all nursing and midwifery staff were on duty and present on the day of facility visit. Of those, interviewers were able to interview approximately 80%, resulting in a total sample size of 1142 (per facility: mean = 2.2, sd = 1.6, min = 1, max = 11). In addition to the motivation scale, the survey contained questions on training, clinical knowledge, compensation, and working conditions. Data was collected on paper and digitalized using a double data entry strategy. Table 2 shows the sample distribution on key characteristics.

**Table 2:** Sample characteristics

Variable	Number	Per cent	Mean	SD	Median	Min	Max
Sex							
Female	646	56.6					
Male	496	43.4					
Age			34.4	5.4	33.5	20	56
Seniority (years in healthcare service)			6.2	5.0	4.5	0	36
<5 years	504	44.1					
≥5 years	638	55.9					
Health worker type							
Nurse/Midwife (diploma)	495	43.4					
Nurse/Midwife (assistant)	647	56.6					
Total	1142	100.0					

Legend: “Nurse/Midwife (diploma)” includes the following cadres: Attaché de santé (specialist nurse), Infirmier Diplômé d’Etat (nurse with state diploma), Sage-Femme/Maïeuticien d’Etat (midwife with state diploma), and Infirmier Breveté (licensed nurse); “Nurse/Midwife (assistant)” includes the following cadres: Accoucheuse Auxilliare (assistant midwife) and Accoucheuse Brevetée (licensed midwife)

### Structural validity analyses

The structural validity analyses (research question 1 (RQ1)) aimed to confirm that the scale measures the motivation dimensions of the SDT continuum as intended. We first conducted a thorough integrated semantic and psychometric item analysis (including inspection of item distribution and correlation patterns; in Stata 12), in response to which we excluded 8 items from the initial 26-item scale due to suboptimal psychometric properties or phrasing (see Additional file 2). The remaining 18 items were subsequently subjected to a confirmatory factor analysis using structural equation modeling (SEM). In line with standard SEM terminology, we refer to the five motivation dimensions as “factors” from here forward. We tested the five-factor model corresponding to Fig. 1 against the three theoretically viable alternative models in Table 3, which emerged as alternative taxonomies during the scale development process or have shown good model-data fit in previous research (e.g., [18, 31]). All modeling was performed with Mplus 7.31, using a maximum likelihood estimator with robust standard errors to account for our non-normal data distribution. Standard errors were adjusted according to the clustered sample structure. Missings were handled with Mplus’ standard full information procedure. All factors were allowed to covary. No cross-loadings or correlated item residuals were specified to facilitate interpretation in light of potential use of the scale with composite scores. Models were evaluated with standard fit indices, including *χ* ^2^, comparative fit index (CFI), standardized root mean square residual (SRMR), and root mean square error of approximation (RMSEA), and compared to each other with the Akaike information criterion (AIC) [32]. For the best-fitting model C, we inspected all model parameters, Mplus’ modification indices, factor correlations, and Cronbach’s *α*. We eliminated 3 further items in the model-fitting process (see Additional file 2), arriving at the final 15-item scale in Table 4. All results presented in this article are based on this final 15-item scale.

**Table 3:** Alternative models tested

Model A	Five-factor model corresponding to Fig. 1
Model B	Four-factor model, combining the integrated and identified types of regulation which have proven difficult to separate in previous research
Model C	Five-factor model as Model B but dividing external regulation into a social and an economic subfactor
Model D	Two-factor model, differentiating autonomous (intrinsic motivation, integrated/identified regulation; AUT) and controlled (introjected, external regulation; CTRL) motivation

**Table 4:** Final item list and descriptive statistics

		Item	Number	Mean	sd	p50	Max	Min
Intrinsic motivation (IM)	im1	Parce que j’aime faire ce que je fais chaque jour au travail. *Because I enjoy doing what I do at work every day.*	1 139	7.91	2.49	9	10	0
	im2	Parce que mes tâches au travail me plaisent beaucoup. *Because I enjoy my work tasks.*	1 142	8.21	2.09	9	10	0
	im3	Parce que le travail que je fais est très intéressant. *Because the work that I do is very interesting.*	1 139	8.22	2.09	9	10	0
Integrated/identified regulation (IDEN)	iden1	Parce qu’être un agent de santé est un élément fondamental de ce que je suis. *Because being a health worker is a fundamental part of who I am.*	1 134	8.08	2.29	9	10	0
	iden2	Parce que mon travail est extrêmement important pour mes patients. *Because my work is extremely important for my patients.*	1 137	8.53	1.89	9	10	0
	iden3	Parce que je veux changer quelque chose dans la vie des autres. *Because I want to make a difference in people’s lives.*	1 138	7.90	2.55	9	10	0
Introjected regulation (INTRO)	intro1	Pour avoir une bonne opinion de moi-même. *In order to feel good about myself.*	1 141	7.45	2.70	8	10	0
	intro2	Parce que ma réputation dépend de mon travail. *Because my reputation depends on my work.*	1 133	7.19	3.00	8	10	0
External regulation-social (EXT-S)	ext1	A cause de la reconnaissance que je reçois de mes patients et de la communauté. *Because of the appreciation I receive from my patients and the community.*	1 132	6.32	3.21	7	10	0
	ext2	Pour ne pas laisser tomber mon équipe. *So I do not let my team down.*	1 136	4.86	3.18	5	10	0
	ext3	Parce que mon responsable direct reconnaît mon travail et m’apprécie. *Because my supervisor recognizes and appreciates me.*	1 128	6.22	3.17	7	10	0
External regulation-economic (EXT-E)	ext4	A cause des avantages liés à mon travail. *Because of the benefits that come with my job.*	1 137	3.75	3.29	4	10	0
	ext5	Pour pouvoir subvenir aux besoins de ma famille. *In order to be able to provide for my family.*	1 141	6.50	3.03	7	10	0
	ext6	Parce que mon travail me procure la sécurité financière. *Because of the financial security my job provides me with.*	1 136	4.76	3.10	5	10	0
	ext7	Afin de gagner de l’argent. *In order to earn money.*	1 134	3.67	3.17	3	10	0

Legend: The English translation is intended to facilitate understanding for the non-French-speaking readership. It is not tested and validated and might thus not be perfectly equivalent to the French version

### Generalizability analyses

The generalizability analyses aimed to confirm that the scale measures the same motivation dimensions equally well in different sample subgroups (“measurement invariance”; RQ2). This is a necessary requirement for later substantive analyses aiming to compare motivation across different health worker subgroups. Specifically, we tested the scale for invariance across sexes, seniority levels, and qualification levels. Following the steps outlined in Table 5 [33], Model C was simultaneously estimated in each respective subgroup, with an increasing number of parameters restricted to equality between subgroups in each testing step. The scale is a measurement invariant at each level when the added equality restrictions do not lead to significantly worse model fit compared to the respective less restricted model. Nested model comparisons were conducted with the rescaled likelihood ratio test [34].

**Table 5:** Measurement invariance testing steps [33]

Test for	Interpretation	Model constraints
Configural invariance	Tests for the assumption of the same underlying factor structure in all subgroups, i.e., the overall model fits the data similarly well in all subgroups	No specific constraints are imposed on the estimated parameters.
Metric invariance	Tests whether the same constructs are measured across subgroups, i.e., whether respondents in different subgroups attribute the same meaning to the respective motivation factors	• Factor loadings estimated freely but constrained to equality in the subgroups
Scalar invariance	Tests whether subgroups can be compared on their mean scores or whether subgroups score systematically different (at the same underlying level of motivation) for certain items	• Factor loadings estimated freely but constrained to equality in the subgroups • Item intercepts estimated freely but constrained to equality in the subgroups
Residual variance invariance	Tests whether the proportion of contamination by other constructs as measured by the different items (i.e., variance that is not explained by the intended factors) is equal across groups and whether measurements are thus fully comparable across groups	• Factor loadings estimated freely but constrained to equality in the subgroups • Item intercepts estimated freely but constrained to equality in the subgroups • Item residual variances estimated freely but constrained to equality in subgroups

### Convergent/discriminant validity analyses

The convergent/discriminant validity analyses aimed to provide further evidence that the scale measures the SDT taxonomy as intended by relating motivation with constructs for which relationships with the SDT motivation dimensions are relatively well established (RQ3). If the new scale does indeed measure what it is intended to measure, relationships with external constructs should approximately correspond to those found in previous research, contextual differences taken into consideration. Specifically, we related motivation to organizational support, organizational commitment, and intentions to quit. Details on hypotheses and measurement of the external constructs are provided in Table 6. We built a separate model for each external construct by adding a measurement model for the respective construct to Model C, allowing the external construct factor to covary with all five motivation factors.

**Table 6:** Convergent/discriminant validation constructs and hypotheses (based on SDT and previous research [15, 18, 43, 44])

Construct and hypotheses	Measurement
Organizational support: extent to which respondents feel supported by their supervisor and coworkers, both technically and emotionally. Hypotheses: (a) Autonomous (intrinsic) types of motivation are closely and positively related to organizational support. (b) Controlled (extrinsic) types of motivation are unrelated to organizational support.	Organizational support was measured with six items partly adapted from [45, 46] (Cronbach’s *α* = .90) Item examples: “The people I work with are there to help me when I need support.”; “I can absolutely rely on the people I work with.” Response scale: 0 (do not agree at all)–10 (completely agree) with visual aid (analogous to the motivation measure)
Organizational commitment: extent to which respondent are emotionally attached to their workplace Hypotheses: (a) Autonomous types of motivation are closely and positively related to organizational commitment. (b) Controlled types of motivation are unrelated to organizational commitment.	Organizational commitment was measured with three items partly adopted from [13, 47] (*α* = .74) Item examples: “I would not want to work for a different health facility.”; “I am proud to be working for this health facility.” Response scale: 0 (do not agree at all)–10 (completely agree) with visual aid (analogous to the motivation measure)
Intentions to quit: extent to which respondents would like to leave their current position Hypotheses: (a) Autonomous types of motivation are negatively related to turnover intentions. (b) Controlled types of motivation are positively related to turnover intentions.	Intentions to quit were measured with three items partly adopted from [11] (*α* = .72) Item examples: “I often feel like leaving my job.”; “Accepting to work for this facility was a mistake.” Response scale: 0 (do not agree at all)–10 (completely agree) with visual aid (analogous to the motivation measure)

## Results

### Structural validity

The structural validity analyses aimed to confirm that the scale does indeed measure the different motivation dimensions of the SDT continuum. We intended to test the “pure” SDT model (Fig. 1; Model A in Table 3) against three theoretically viable alternative models. Unfortunately, Model A could not be estimated with the final subset of items as we were only able to retain one integrated regulation item. Table 7 presents fit statistics for the three alternative models. Model C, which combines the integrated and identified dimensions but differentiates external regulation into a social and an economic subcomponent, clearly demonstrated the best fit. *χ* ^2^ was significant as expected given our relatively large sample size, high factor correlations, and non-normally distributed data [32, 35] but of a magnitude that does not warrant concerns for model fit. All other fit indices were good in absolute terms, indicating that the modified five-factor model is well represented in the data. All following results thus pertain to Model C. A graphic representation including standardized coefficients for all estimated parameters as well as modification indices is given in Additional file 2. For each motivation factor, item-factor loadings are of relatively similar magnitude; the items thus indicate the respective factor with similar strength. Modification indices signal that some items, particularly ext6 and ext7, load on factors other than the intended to some extent. Overall, however, such cross-loadings are low in magnitude, indicating good item discriminatory power. Although also mostly low in magnitude, modification indices show many residual (error term) correlations, particularly for the external regulation (EXT) items. Factor correlations (Table 8) display the expected simplex pattern, i.e., decreasing magnitude with decreasing conceptual closeness. Cronbach’s *α* is relatively low for all factors.

**Table 7:** Results of the structural validation analyses

Model		*χ* ^2^	df	*p*	RMSEA	*p* RMSEA ≤.05	CFI	SRMR	AIC
A	Model A, the original five-factor model corresponding to Fig. 1, could not be estimated as only one integrated regulation item was retained in the fitting process (at least two are necessary for model identification)								
B	Four-factor model: IM (im1-im3), IDEN (iden1-iden3), INTRO (intro1 intro2), EXT (ext1-ext7)	472	84	.000	.064	.000	.867	.069	78 649
C	Five-factor model: IM (im1-im3), IDEN (iden1-iden3), INTRO (intro1 intro2), EXT-S (ext1-ext3), EXT-E (ext4-ext7)	227	80	.000	.040	.996	.950	.033	78 318
D	Two-factor model: AUT (im1-iden3), CTRL (intro1-ext7)	677	89	.000	.076	.000	.799	.076	78 927

Interpretation of fit indices [32]: Insignificant *χ* ^2^ values indicate good model-data fit. However, due to a number of conceptual and statistical issues, *χ* ^2^ is often significant even in the case of a relatively good model fit. CFI values approaching .95 as well as RMSEA values of .05 or smaller and SRMR values of .05 and smaller are considered indicative of good model fit. Smaller AIC values indicate better data-model fit compared to alternative models (evaluation goodness of fit (likelihood function) versus complexity of the model)

Legend: *IM* intrinsic motivation factor, *IDEN* integrated/identified regulation factor, *INTRO* introjected regulation factor, *EXT* external regulation factor, *EXT-S* external regulation-social factor, *EXT-E* external regulation-economic factor, *AUT* autonomous motivation factor, *CTRL* controlled motivation factor

**Table 8:**
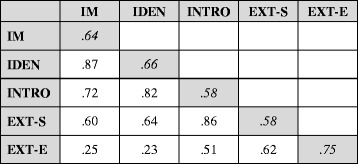
Model-estimated factor correlation matrix and Cronbach’s *α* (on the shaded diagonal cells) for the motivation factors in Model C

All correlation coefficients are Person correlations and significantly different from zero

Legend: *IM* intrinsic motivation, *IDEN* integrated/identified regulation, *INTRO* introjected regulation, *EXT-S* external regulation-social, *EXT-E* external regulation-economic

### Generalizability

The measurement invariance analyses aimed to confirm that the scale has the same measurement properties in different subsamples and that measurements (scores, variances, etc.) can thus be compared between health worker subgroups. Table 9 shows the results for sex, seniority, and health worker qualification level. The scale is fully invariant for seniority in healthcare. Only partial measurement invariance could be established for sex. Specifically, women scored higher than men on intro1 and ext6, but lower on intro2, at the same underlying levels of introjected and external regulation, respectively (scalar non-invariance). This raises concerns about factor means comparability for the concerned subscales. However, as intro1 and intro2 are biased in opposite directions in around the same magnitude, we can assume biases to cancel each other out. For ext6, considering that it is only one of four items measuring economic external regulation and the systematic difference in scoring is relatively small, we can also assume that the overall bias is of little practical relevance [33]. We could also establish only partial scalar invariance for qualification level. Item ext7 had a somewhat higher factor loading (i.e., item is more strongly indicative of factor) in fully qualified than in assistant nurses (metric non-invariance). At the scalar level, fully qualified nurses systematically scored higher on intro1, ext7, and im3 and lower on intro2. In similar lines of reasoning as for sex, we can reasonably assume that these systematic differences do not majorly threaten comparability between groups substantially, however.

**Table 9:** Measurement invariance testing results

		Absolute model fit							Likelihood ratio test info and results			
		*χ* ^2^	df	*p*	RMSEA	*p* RMSEA ≤.05	CFI	SRMR	#free parms	LR (with model above)	df	*p* (.05)
	Baseline model C	227	80	.000	.040	.996	.950	.033	–	–	–	–
Sex	Configural invariance	333	160	.000	.044	.994	.943	.041	110	–	–	–
	Metric invariance	344	170	.000	.042	.975	.943	.043	100	9.40	10	0.50
	Scalar invariance	386	180	.000	.045	.917	.932	.045	90	50.44	10	< 0.01
	Scalar invariance, partial	356	178	.000	.042	.983	.941	.044	92	11.19	8	0.19
	Residual variance invariance	369	191	.000	.040	.995	.941	.048	79	16.30	13	0.23
Seniority	Configural invariance	332	160	.000	.043	.950	.943	.039	110	–	–	–
	Metric invariance	342	170	.000	.042	.979	.943	.042	100	9.23	10	0.51
	Scalar invariance	350	180	.000	.041	.993	.944	.043	90	4.29	10	0.93
	Residual variance invariance	366	195	.000	.039	.998	.944	.052	75	19.39	15	0.20
Qualification level	Configural invariance	319	160	.000	.042	.980	.947	.039	110	–	–	–
	Metric invariance	338	170	.000	.042	.984	.945	.044	100	18.43	10	0.05
	Scalar invariance	371	180	.000	.043	.966	.937	.046	90	37.07	10	< 0.01
	Scalar invariance, partial	349	177	.000	.041	.989	.943	.045	93	9.26	7	0.25
	Residual variance invariance	363	192	.000	.040	.998	.944	.048	78	18.91	15	0.22

Legend: Interpretation of the absolute model fit indices [32]: Insignificant *χ* ^2^ values indicate good model-data fit. However, due to a number of conceptual and statistical issues, *χ* ^2^ is often significant even in the case of relatively good model fit. CFI values approaching .95 as well as RMSEA values of .05 or smaller and SRMR values of .05 and smaller are considered indicative of good model fit

Interpretation of the likelihood ratio test statistics: #free parms is the number of freely estimated model parameters; these are gradually restricted in the invariance testing process as parameters are forced to equality in the compared subgroups (see Table 5). LR (with above model and its degrees of freedom) is the *χ* ^2^-distributed test statistic of the rescaled likelihood ratio test. In each row, it refers to the difference in fit of the respective model and the next less restrained (i.e., above) model. Statistical insignificance indicates that the more restricted model fits similarly as the above less restricted model, i.e., that the added parameter equality restrictions for the compared sample subgroups do not substantially worsen model fit and that the scale can thus be considered measurement invariant for the compared groups at the respective level

### Convergent/discriminant validity

The convergent/discriminant validity analyses aimed to provide additional evidence that the scale measures what it was intended to measure by relating motivation to other variables with which the relationship is well established. Table 10 shows correlations of the motivation factors with the three constructs introduced in Table 6. Correlation patterns are generally in the expected directions, supporting the notion that the scale measures the SDT continuum of motivation well. Organizational support and organizational commitment are more strongly related to introjected regulation than expected based on previous research. Correlations of all motivation factors with intentions to quit are weaker than expected. These findings are likely substantive findings reflecting realities in the specific context rather than being indicative of measurement issues, however [6].

**Table 10:** Convergent/discriminant validation results: model-estimated factor correlations of motivation dimensions with external constructs

	IM	IDEN	INTRO	EXT-S	EXT-E
Organizational support	.46	.43	.37	.47	.12
Organizational commitment	.58	.54	.37	.38	.05^a^
Intentions to quit	−.15	−.07^a^	.06^a^	.03^a^	.18

Legend: ^a^not statistically significantly different from zero

## Discussion

The paper presents evidence on the validity of a newly developed scale to measure motivation composition of health workers, i.e., the relative contribution of different kinds of motivation to their overall work motivation, from a sample of nurses in Burkina Faso.

**Fig. 2:**
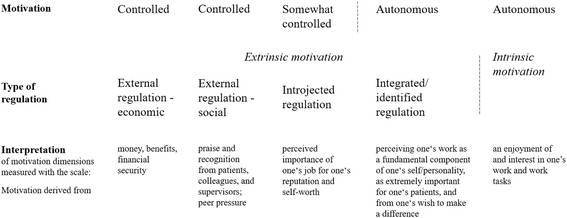
The modified SDT taxonomy of motivation as measured by the scale

Our findings show that the scale measures a somewhat modified version of the SDT continuum of motivation well and relatively consistent in different health worker subgroups. Specifically, our analyses suggest that the scale is not able to distinguish between integrated and identified regulation. This finding is in line with what emerged during the scale development process and with previous attempts to measure the SDT continuum [18, 29]. From an applied perspective, not distinguishing the two dimensions is even advantageous insofar as policy implications are similar and interpretation thus facilitated. Our analyses further suggest to separate external regulation into a social dimension, including aspects of social interaction and recognition, and an economic dimension, pertaining to the economic security one’s job provides. Again, such a distinction is sensible from an applied point of view in light of the different policy implications related to the two dimensions. The modified taxonomy measured by the scale is visualized in Fig. 2.

### Methodological discussion

Our results are generally as expected. The structural and convergent/discriminant validity analyses support that the scale measures the SDT taxonomy of motivation, albeit in slightly modified form as explained above. It does so equally well for different health worker subgroups, although with some caveats (see below), indicating that the scale can be used for between-group comparisons.

However, two aspects deserve further discussion. First, despite good overall fit of the data to the five-factor model, we found relatively low levels of Cronbach’s *α* for all factors but EXT-E. While low *α*s are no longer perceived as indicators of low measurement quality [36–38], they do signal that our items cover different sub-aspects of the respective dimensions rather than being extremely similar. This is no problem per se, but the relative conceptual breadth of the motivation dimensions should be taken into account when interpreting measurements. Should *α* be even lower in other settings, a re-evaluation of the scale items and the scale’s dimensionality might be necessary. Second, factor correlations were relatively large in magnitude compared to other SDT-based measures (e.g., [18]). We believe there to be two main reasons: Respondents’ generally scored relatively high despite the various measures in place, the common method and acquiescence bias likely inflating correlations [39]. Additionally, we found cross-loadings and residual correlations for many items, which, although mostly small, likely also contributed to inflated factor correlations. They might have partially been caused by the more specific item phrasing compared to other SDT-based measures [18, 29]. Cross-loadings and residual correlations are often explicitly modeled to improve overall model fit, for instance, in exploratory structural equation models (ESEM) [32, 40]. In light of our already good fit, we opted against doing so based on the assumption that future users of the scale might want to analyze data using composite scores, which would be difficult with a scale “calibrated” in an ESEM framework.

### Limitations

#### Measurement reliability and sensitivity

We were unable to examine measurement reliability (i.e., accuracy and consistency) in-depth within the scope of our study, beyond what was possible in the scale development process. We thus cannot exclude that respondents’ scores are to some extent influenced by random or systematic measurement error rather than solely by underlying levels of motivation. The convergent/discriminant validity analysis results, specifically their consistency with previous research, imply that random measurement error is at acceptable levels. Based on the continued high scores on many items, however, we suspect that some social desirability or acquiescence bias might still be at play, systematically inflating scores in relation to their “true values” for certain items. This warrants caution when interpreting absolute scores and calls into question the scale’s sensitivity “at the ceiling,” i.e., its ability to distinguish respondents or measure change at high motivation levels. Generally, note that systematic biases are less of a concern when investigating relationships of motivation with other variables or changes in motivation over time, assuming that biases stay constant.

#### Criterion validity

In addition to the convergent/discriminant validity analyses in this study, it would be important to also examine the scale against more tangible criteria such as work performance in the future.

### Recommendations for future use

We welcome the use of the scale in future research and are confident that the scale will prove a valid instrument with health workers in other countries and settings as well. The scale will be useful for researchers who want to not only investigate overall levels of work motivation (“motivation intensity”) but also study how motivation of different origin and characteristics contribute to these overall levels (“motivation composition”) to understand how different “motivation profiles” relate to outcomes of interest [4].

Based on our experiences with the scale so far, we would like to offer the following recommendations to researchers interested in using the tool:

#### Use the full 26-item scale

Use the full 26-item scale, if possible within the scope of your research. Although we are confident that the item list covers the most important reasons for work motivation even beyond Burkinabé nurses, our item selection for the final 15-item scale was heavily empirically driven and thus reliant on the specific sample. We cannot exclude that a different item selection would have resulted from a different sample.

#### Use a response scale with seven to nine options

Although our 11-point scale seemed to have had certain advantages, we suspect that it might have overwhelmed some respondents, who might have had difficulty conceptualizing the fine differences between scores on the 11-point scale. See Additional file 1 for a more extensive discussion.

#### Test for measurement invariance

Test for measurement invariance to identify non-invariant scale items before moving on to the actual analysis of interest. Our generalizability analyses suggest that it is possible to compare measurements for different health worker subgroups on all statistical parameters (e.g., means, variances) if analyses are performed in an SEM framework. If factor means for different subgroups are to be compared using composite scores, however, systematic differences in scoring between groups are potentially more problematic as they might artificially create non-real or mask real group differences [37, 41]. In our sample, respondents from different subgroups showed somewhat different scoring behavior on items im3, intro1, intro2, ext6, and ext7.

#### Use SEM for the actual analysis of interest

Generally, substantive analyses on data collected with the scale can be done in one of the following two ways: One can either calculate composite scores or use them in any other type of analysis (e.g., predictor or outcome variables in regression models). Composite scores are usually calculated as the unweighted means of responses to all items pertaining to a factor/dimension. Alternatively, one can continue in an SEM framework by adding a structural part corresponding to a regression model to the measurement model. The composite score calculation is skipped and substantive relationships are directly estimated from the items via the latent factors, thus preserving full variance in the data. For this and other reasons, SEM is clearly preferred by psychometricians and generally leads to better estimates [37, 42] but is statistically complex and requires large samples [32].

Beyond its general advantages, we also recommend SEM based on a number of specific results of our analyses. Calculating composite scores bears a risk of imprecision if systematic differences in item-factor loadings (i.e., items have different indicative values for the motivation factor) or intercepts (i.e., systematic differences in item scores which are unrelated to the underlying motivation level) are not accounted for. As with other biases, this is less of an issue if relationships between variables or change over time is the focus of interest, but of critical importance if interpretation of absolute motivation levels is planned. We found only slightly inhomogeneous factor loadings and intercepts in our sample which did not seem to lead to substantial differences between composite scores and latent factor scores. However, more substantial differences are possible in other settings. If the use of SEM is not feasible, we strongly recommend developing a good understanding of all item properties before embarking on substantive analyses with composite scores. Should differences in factor loadings or intercepts across items be more substantial, one might consider weighing items when calculating composite scores rather than giving equal weight to all items, or adding constants to balance differences in intercepts. Note that such adjustments have implications for the interpretation of the measurement (i.e., the “meaning” and level of the respective motivation dimension), depending on how each item effectively contributes to the composite scores. They should thus be applied with caution.

## Conclusions

This article presents evidence for the validity of a Self-Determination Theory-based scale to measure health worker motivation composition. Our results show that the scale measures a modified version of the SDT taxonomy well and relatively consistently across health worker subgroups. Results of the convergent/discriminant validation indicate that the five dimensions of motivation relate differently to important work outcomes, underlining the value of investigating motivation composition for the development of a more profound understanding of health worker motivation. We hope that our tool will contribute to meaningful research informing the design of effective and side effect-free interventions to enhance motivation and performance.

## Additional files

Additional file 1: Development process of a new SDT-based motivation composition measure in Burkina Faso. (DOCX 287 kb)

Additional file 2: Items eliminated in the analytical process, standardized parameter estimates for Model C, and suggested modification indices for Model C. (DOCX 107 kb)

## Abbreviations

AIC: Akaike information criterion
AUT: Autonomous motivation
CFI: Comparative fit index
CTRL: Controlled motivation
ESEM: Exploratory structural equation modeling
EXT: External regulation
EXT-E: External regulation-economic subcomponent
EXT-S: External regulation-social subcomponent
IDEN: Integrated/identified regulation
IM: Intrinsic motivation
INTRO: Introjected regulation
LMIC: Low- and middle-income country
PBF: Performance-based financing
RMSEA: Root mean square error of approximation
RQ: Research question
SDT: Self-Determination Theory
SEM: Structural equation modeling
SRMR: Standardized root mean square residual

## References

[1] Campbell J, Dussault G, Buchan J, Pozo-Martin F, Guerra Arias M, Leone C (2013). A universal truth: no health without a workforce. Forum report, Third Global Forum on Human Resources for Health, Recife, Brazil.

[2] Meessen B, Soucat A, Sekabaraga C (2011). Performance-based financing. Just a donor fad or a catalyst towards comprehensive health-care reform?. Bull World Health Organ.

[3] Dieleman M, Gerretsen B, van der Wilt GJ (2009). Human resource management interventions to improve health workers’ performance in low and middle income countries. A realist review. Health Res Policy Syst.

[4] Lohmann J, Houlfort N, De Allegri M (2016). Crowding out or no crowding out? A Self Determination Theory approach to health worker motivation in performance-based financing. Soc Sci Med.

[5] Renmans D, Holvoet N, Orach CG, Criel B (2016). Opening the ‘black box’ of performance-based financing in low- and lower middle-income countries: a review of the literature. Health Policy Plan.

[6] Willis-Shattuck M, Bidwell P, Thomas S, Wyness L, Blaauw D, Ditlopo P (2008). Motivation and retention of health workers in developing countries. A systematic review. BMC Health Serv Res.

[7] Bertone MP, Witter S (2015). The complex remuneration of human resources for health in low-income settings: policy implications and a research agenda for designing effective financial incentives. Hum Resour Health.

[8] Henderson LN, Tulloch J (2008). Incentives for retaining and motivating health workers in Pacific and Asian countries. Hum Resour Health.

[9] Pinder CC (2008). Work motivation in organizational behavior.

[10] Franco LM, Bennett S, Kanfer R, Stubblebine P (2004). Determinants and consequences of health worker motivation in hospitals in Jordan and Georgia. Soc Sci Med.

[11] Bennett S, Franco LM, Kanfer R, Stubblebine P (2001). The development of tools to measure the determinants and consequences of health worker motivation in developing countries.

[12] Prytherch H, Leshabari MT, Wiskow C, Aninanya GA, Kakoko DCV, Kagoné M (2012). The challenges of developing an instrument to assess health provider motivation at primary care level in rural Burkina Faso, Ghana and Tanzania. Glob Health Action.

[13] Mbindyo PM, Blaauw D, Gilson L, English M (2009). Developing a tool to measure health worker motivation in district hospitals in Kenya. Hum Resour Health.

[14] Deci EL, Ryan RM (1985). Intrinsic motivation and self-determination in human behavior.

[15] Gagné M, Deci EL (2005). Self-determination theory and work motivation. J Organ Behav.

[16] Deci EL, Ryan RM (2000). The ‘what’ and ‘why’ of goal pursuits: human needs and the self-determination of behavior. Psychol Inq.

[17] Miquelon P, Vallerand RJ (2008). Goal motives, well-being, and physical health: an integrative model. Can Psychol.

[18] Gagné M, Forest J, Vansteenkiste M, Crevier-Braud L, van den Broeck A, Aspeli AK (2015). The multidimensional work motivation scale: validation evidence in seven languages and nine countries. Eur J Work Organ Psychol.

[19] Paul F (2009). Health worker motivation and the role of performance based finance systems in Africa. A qualitative study on health worker motivation and the Rwandan performance based finance initiative in district hospitals. Master’s thesis.

[20] Chandler CIR, Chonya S, Mtei F, Reyburn H, Whitty CJM (2009). Motivation, money and respect: a mixed-method study of Tanzanian non-physician clinicians. Soc Sci Med.

[21] Dieleman M, Cuong PV, Anh LV, Martineau T (2003). Identifying factors for job motivation of rural health workers in North Vietnam. Hum Resour Health.

[22] Dieleman M, Toonen J, Touré H, Martineau T (2006). The match between motivation and performance management of health sector workers in Mali. Hum Resour Health.

[23] Peters DH, Chakraborty S, Mahapatra P, Steinhardt L (2010). Job satisfaction and motivation of health workers in public and private sectors: cross-sectional analysis from two Indian states. Hum Resour Health.

[24] Goldberg AB, Ron LI (2012). Understanding the complex drivers of intrinsic motivation for health workers in Malawi. Health Systems 20/20 project report.

[25] Morrison J, Batura N, Thapa R, Basnyat R, Skordis-Worrall J (2015). Validating a tool to measure auxiliary nurse midwife and nurse motivation in rural Nepal. Hum Resour Health.

[26] Ministère de la Santé du Burkina Faso (2015). Annuaire Statistique 2014.

[27] Codjia PL, Ouoba V (2003). Motivation, leadership et performance des équipes dirigeantes du secteur public de la santé du Burkina Faso.

[28] Scheewe S, Dieleman M, Millogo JJ, Traore A (2013). Planification pour une couverture universelle: les ressources humaines en santé maternelle, néonatale et infantile au Burkina Faso en 2013-2025.

[29] Tremblay MA, Blanchard CM, Taylor S, Pelletier LG, Villeneuve M (2009). Work Extrinsic and Intrinsic Motivation Scale. Its value for organizational psychology research. Can J Behav Sci.

[30] Institute of Public Health Heidelberg University, The World Bank, Ministère de la Santé, Centre Muraz, Centre Hospitalier de l’Université de Montréal (2015). Impact evaluation for health performance-based financing in Burkina Faso.

[31] Vansteenkiste M, Lens W, De Witte S, De Witte H, Deci EL (2004). The ‘why’ and ‘why not’ of job search behaviour: their relation to searching, unemployment experience, and well-being. Eur J Soc Psychol.

[32] Kline RB (2010). Principles and practice of structural equation modeling. 3rd revised edition.

[33] Vandenberg RJ, Lance CE (2000). A review and synthesis of the measurement invariance literature: suggestions, practices, and recommendations for organizational research. Organ Res Methods.

[34] Bryant FB, Satorra A (2012). Principles and practice of scaled difference chi-square testing. Struct Equ Model..

[35] Fan X, Thompson B, Wang L (1999). Effects of sample size, estimation method, and model specification on structural equation modeling fit indexes. Struct Equ Model.

[36] Cortina JM (1993). What is coefficient alpha? An examination of theory and applications. J Appl Psychol.

[37] Borsboom D (2006). The attack of the psychometricians. Psychometrika.

[38] Sijtsma K (2009). On the use, the misuse, and the very limited usefulness of Cronbach’s alpha. Psychometrika.

[39] Podsakoff PM, MacKenzie SB, Lee JY, Podsakoff NP (2003). Common method biases in behavioral research: a critical review of the literature and recommended remedies. J Appl Psychol.

[40] Marsh HW, Morin AJ, Parker PD, Kaur G (2014). Exploratory structural equation modeling: an integration of the best features of Exploratory and Confirmatory Factor Analysis. Annu Rev Clin Psychol.

[41] Steinmetz H (2013). Analyzing observed composite differences across groups: is partial measurement invariance enough?. Meth Eur J Res Meth Behav Soc Sci.

[42] Skrondal A, Laake P (2001). Regression among factor scores. Psychometrika.

[43] Gillet N, Gagné M, Sauvagère S, Fouquereau E (2013). The role of supervisor autonomy support, organizational support, and autonomous and controlled motivation in predicting employees’ satisfaction and turnover intentions. Eur J Work Organ Psychol.

[44] van den Broeck A, Howard J, Leroy H, Gagné M. Motivated to work: a meta-analysis of regulations. Conference presentation at the 6th International Conference on Self-Determination Theory, Victoria, Canada; 2016.

[45] Baruch-Feldman C, Brondolo E, Ben-Dayan D, Schwartz J (2002). Sources of social support and burnout, job satisfaction and productivity. J Occup Health Psychol.

[46] Eisenberger R, Huntington R, Hutchison S, Sowa D (1986). Perceived organizational support. J Appl Psychol.

[47] Boardman C, Sundquist E (2009). Toward understanding work motivation. Worker attitudes and the perception of effective public service. Am Rev Public Adm.

