# Supplementary material for: Measuring health workers’ motivation composition: validation of a scale based on Self-Determination Theory in Burkina Faso
Source: Hum Resour Health. 2017 May 22;15:33. doi: 10.1186/s12960-017-0208-1 (PMC5441099; doi:10.1186/s12960-017-0208-1)
Supplement: Supplementary file 1 — Development process of a new SDT-based motivation composition measure in Burkina Faso. (DOCX 287 kb) [file 12960_2017_208_MOESM1_ESM.docx]

**Development process of a new SDT-based motivation composition measure in Burkina Faso**

Contents

[1. SDT-based measurement of motivation 2](#_Toc473277650)

[2. Rationale for development of a new measure, and key experience-based design decisions 2](#_Toc473277651)

[3. Item development process 4](#_Toc473277652)

[4. Pretest 4](#_Toc473277653)

[5. Final motivation composition measure used in the validation study 9](#_Toc473277654)

[6. To what extent were the design decisions based on the Malawi experience successful in counteracting challenges encountered in Malawi? 14](#_Toc473277655)

[7. References 17](#_Toc473277656)

1. **SDT-based measurement of motivation**

The SDT-based literature has generated a number of self-report measures of self-determined motivation over the years, in an occupational context for instance the Work Extrinsic and Intrinsic Motivation Scale (WEIMS; [1]) or the Multidimensional Work Motivation Scale (MWMS; [2]). Common to these measures is the idea that individuals reveal their locus of causality for behavior (“latent variables”) in the reasons for their actions they provide (“manifest variables”) [3]. Following a guiding question such as “Why do you do your work?” (WEIMS) or “Why do you or would you put efforts into your current job?” (MWMS), a list of reasons pertaining to the five types of motivation, such as “Because I have fun doing my job.” (MWMS, intrinsic motivation) or “Because it has become a fundamental part of who I am.” (WEIMS, integrated regulation), is provided. Participants are asked to indicate the extent to which these reasons correspond to those for which they personally are involved in their work. The newly developed measurement tool validated in this paper follows the same measurement rationale.

1. **Rationale for development of a new measure, and key experience-based design decisions**

We decided to develop a new measure rather than using an existing measure of the SDT continuum of motivation based on our experiences from an attempt to apply the WEIMS [1] in Malawi within the context of a previous similar study [4]^[[1]](#footnote-1)^. We drew a number of lessons from this experience which informed the development of the new scale in Burkina Faso. The two most important, pertaining to respondents’ ability to process abstract items and to respondents’ scoring patterns, are summarized in Table 1.

| Main challenges encountered in Malawi | Design decisions to address the challenges in Burkina Faso |
| --- | --- |
| Health workers struggled with high level of abstraction required by items and guiding question of the WEIMS | 1. More specific item phrasing tailored to the health care delivery context and the social service nature of health care provision 2. Direct phrasing of guiding question, asking participants for the importance of different reasons for motivation at work to them personally rather than to indicate their degree of agreement with statements |
| We experienced strong social desirability and acquiescence bias reflected in very high scores which did not seem to reflect respondents‘ true feelings as expressed in discussions outside the official data collection, but rather local norms to respond positively. This resulted in little variation in the data using a standard 5-point answer scale. | 1. Use of an 11-point answer scale rather than a 5-point scale, with visual aid (see section 5), to allow health workers to express variation in their answers while permitting adherence to ‘positivity norms’ 2. Framing exercise to help respondents anchor their answers in their personal set of work motivations by asking them to reflect on their motivations and the relative importance of different aspects before answering to the items (see section 5) 3. ‘Hybrid’ mode of scale administration: self-entry of answers in separate questionnaire copy in an otherwise face-to-face interview to maximize perceived confidentiality while minimizing risk for poor understanding or acquiescence bias (‘rushing through’) by having the interviewer explain instructions and read out every statement |

1. **Item development process**

We opted to forego an exploratory qualitative pre-study to inform the item design in light of our relative confidence that the SDT taxonomy of motivation is appropriate to an LMIC health care context as discussed in the background section of the paper and our experience in Malawi. Rather, we decided to start the item development process based on the literature [3,5-17] by collecting emerging ‘motivational themes’ and categorizing them according to the SDT taxonomy. Based on this and drawing inspiration from the WEIMS [1] and MAWS [18], we next elaborated between 4 and 12 items per dimension with the intention of selecting a subsample for a short final scale at the analytical stage^[[2]](#footnote-2)^. This preliminary item list was then discussed intensively with local experts^[[3]](#footnote-3)^ including nurses, a medical doctor, and a sociologist to ascertain that the taxonomy is appropriate and useful to capture local sources of motivation and to ensure that all dimensions are well and context-appropriately captured by the items.

1. **Pretest**

We conducted an extensive pretest in July and August 2013 to assess the quality of the newly developed scale and to allow for necessary modifications before implementation in the context of the PBF impact evaluation. In addition to the motivation measure, the measures used for the discriminant/convergent validation analysis were also tested.

The pretest aimed at answering the following questions:

- Content validity: To what extent does the tool reflect all important aspects of the constructs to be studied in the respective context?
- Item quality: Are items and answer scales unambiguous, easy to understand? Is there any proneness to biased answers?
- General tool quality: To what extent is the tool practical to administer? Will providers be able to keep up their concentration and motivation? Is a French survey sufficient, or will translation into local languages be necessary?
- Initial construct validity: Does the assumed factor structure of the tool hold true empirically?

The pretest consisted of two major elements:

- A pilot pretest of the newly developed questionnaire section on selected health workers, conducted by trained interviewers, but with members of the research team present and observing and followed by unstructured post-survey interviews of selected health workers by the research team
- A full quantitative pretest on 59 health workers. The pretest was done as a normal, face-to-face interview. We only adopted the hybrid format as described below following the pretest.

**Pretest sample**

The pretest was conducted in a 12-day period with nurses of 17 health facilities in the Health and Demographic Surveillance System (HDSS) area of Nouna district in the Boucle du Mouhoun region. Although part of the PBF implementation, the HDSS area in Nouna was excluded from the impact evaluation as we did not want to subject households to surveys beyond the already heavy burden of the HDSS panel. Health facilities and health workers within the HDSS area can be assumed very similar to other health facilities where our impact evaluation took place, but were available for a pretest without introducing a bias for the main study. Within the 17 facilities, all nurses were interviewed. Interviews were conducted by two interviewers specifically trained for this purpose.

The final sample included 21 nurse/midwives with diploma, and 31 assistant nurse/midwives. Pretest participants had worked as health care providers for an average of 5.3 years (sd= 4.0, min=0, max=20).

**Pretest results**

- Content validity: Expert discussions during the item development process as well as the unstructured post-survey interviews with selected health workers confirmed the validity of the SDT taxonomy in the context: In speaking about different sources of motivation, intrinsic interest in work tasks, reputational and self-worth-related aspects, and external motivators were clearly distinguished. With regard to the latter, social and economic aspects emerged as separate themes. The distinction between integrated and identified regulation was less clear, with feelings of identification with the job appearing to be largely grounded in altruistic notions. At the item level, experts and health workers affirmed that the constructs were well covered, in context-appropriate language.
- General tool quality: There were few practical difficulties in administering the tool. The questionnaire was long, and although health workers seemed able to handle them, a reduction in length of the different psychometric scales would be preferable. Following a few minor changes in wording, Burkinabé health workers had no language difficulties with the French version. Translation into local languages thus seemed unnecessary.
- Item quality: A few changes in wording were made in light of the differences in the use of specific words in France and Burkina Faso, and local terminology (all done before the full quantitative pretest). Following these changes, there were no apparent difficulties in understanding the items. Similarly, there were no apparent difficulties in understanding the answer scales. To ensure proper explanation of answer scales by the interviewers, a standard explanation was drafted for use during training and for reference on the field. We observed that health workers were reluctant to give answers that implied dissatisfaction or a negative judgement of their situation during the pilot pretest. It is unclear whether this was due to the presence of the research team members, or a general issue. However, the analysis of the full quantitative pretest data substantiate that social desirability might have continued to be a challenge.
- Initial construct validity: The pretest data was investigated with standard psychometric procedures, particularly exploratory factor analysis (principal component analysis). The following initial results need to be interpreted with care due to the small sample size. Health workers tended to score rather highly on the 0-11 point scale. The intended factor structure was somewhat reflected in the data, but not ideally. The integrated and identified types were particularly difficult to separate, which is in line with what other authors have reported before [e.g.18]. Internal consistencies for the motivational regulation subscales were not ideal, but at just about acceptable levels.

**Further modifications in response to pretest results**

- A number of items were slightly rephrased to account for minor difficulties in comprehension apparent from the pretest.
- In order to address the challenges of consistently high scoring, the administration format was changed to a hybrid format. The idea was to allow for answer to be given confidentially so as to minimize social desirability bias (as in a self-administered survey), while at the same time ensuring that everything is understood, independently of the respondents’ reading and comprehension skills, and minimizing the risk of ‘rushing through’ the survey (as in face-to-face interview). Specifically, the ‘hybrid sections’ were administered as follows:
  - As in a pure face-to-face interview, the interviewers read our all instructions and explained the response format. They were instructed not to give any additional explanation, but rather stress answers should be about what health workers subjectively understand.
  - Interviewers also read out all reasons (items). They were instructed to allow enough time for the respondent to think and answer before moving onto the next statement.
  - However, contrary to a regular face-to-face interview, respondents also received a questionnaire copy with the items, answer scales, and answer entry boxes. They read along as the interviewers read out the reasons, and entered their answers on their own in a private manner. No answers were given to the interviewer.
  - After completion of the hybrid sections, respondents returned their questionnaires in a sealed envelope. The rest of the survey was conducted in the standard face-to-face format.
- Interviewers were further more explicitly instructed to ensure an interview location with maximum privacy, as well as to stress privacy and confidentiality throughout the survey. Interviewers were also more explicitly instructed to ensure respondents understand the correct use of the answer scale, i.e. that it can and should be used in its entirety, and the 10 should only be chosen for the absolute most important aspects.

The resulting final scales were tested again in the context of the pretest of the impact evaluation baseline data collection in Burkina Faso. This additional pretest demonstrated that the general concept of the tool, the hybrid mode of administration, and the items themselves were well understood and easy to handle. Quantitative analysis of the data resulting from this additional pretest was not possible.

1. **Final motivation composition measure used in the validation study**

**Introductory texts and framing exercise**

Note that the English translation is intended to facilitate understanding for the non-French-speaking readership. It is not tested and validated and might thus not be perfectly equivalent to the French version.

| **French** | **English** |
| --- | --- |
| Cette partie du questionnaire contient des questions au sujet de votre motivation au travail, sur l’environnement de votre travail et sur votre satisfaction par rapport aux différents aspects de votre travail. Je vais vous lire des affirmations que vous pouvez également lire sur votre fiche. Cependant, contrairement à la procédure antérieure, vous ne me direz pas votre réponse, mais vous noterez plutôt vous-même la réponse sur votre fiche. Veuillez prendre votre temps pour décider de votre réponse et me signaler lorsque nous pouvons continuer avec l'affirmation suivante. Une fois que nous aurons terminé, vous mettrez votre fiche dans une enveloppe scellée. De cette façon, vos réponses restent privées. Je vais maintenant vous expliquer comment répondre à ces affirmations. Avez-vous des questions de compréhension ? | This part of the questionnaire contains questions related to your work motivation, to your working environment, and to your satisfaction with different aspects of your work. I will read statements to you that you will also find on your copy of the questionnaire. Unlike before, do not tell me your answer, but please record your answer yourself on your questionnaire copy. Please take your time to decide on your answer and signal to me when you are ready to continue with the next statement. In order for your answers to remain private, I will provide an envelope for your copy of the questionnaire once we’ve completed this part of the interview. I will now explain to you how to respond to the statements you will hear and read. Do you have any questions so far? |
| *The interviewer explains how to use the answer scale* | |
| Pour commencer, je vais vous lire différentes raisons pour lesquelles vous pourriez être motivés à travailler. Certaines raisons pourront vous sembler personnellement importantes, d'autres par contre non. Les individus diffèrent dans la manière dont ils sont motivés à travailler en fonction de leur situation et de leur personnalité. Il est essentiel de comprendre que toutes les raisons que les individus peuvent avoir sont tout aussi valables et qu'aucune n'est «meilleure» ou «pire» qu'une autre. De ce fait, il est très important pour notre recherche que vous répondiez exactement comme vous vous sentez, même si vous pensez que nous pourrions ne pas partager votre avis. | To start out, I will read to you different reasons for which you might be motivated to work. You will find that some are very important to you, while others might be less important for you personally. People differ in their main reasons for being motivated at work, depending on their unique situations and personalities. It is pivotal to understand that all reasons people might have are equally valid, none are 'better' or 'worse' than others. It is very important to our research that you answer exactly as you feel, even if you think that we might not be happy with your answer. |
| Avant de commencer, veuillez prendre une minute pour réfléchir sur les raisons les plus importantes pour lesquelles vous travaillez. | Before we begin, please take a minute and think about the most important reasons for which you work. |
| *The interviewer allows health workers a minute of reflection.* | |
| Commençons! S'il vous plaît, pensez à la manière dont vous vous êtes senti principalement au cours des quatre dernières semaines lors de la réponse. Pour chacune de ces raisons que je vais vous lire, veuillez indiquer si elles sont importantes ou non par rapport aux raisons les plus importantes pour lesquelles vous travaillez. | Let's begin. Please think of how you've predominantly felt in the past four weeks when answering. For each of the following reasons, indicate whether these are important or not important in relation to the most important reasons for which you work. |

**Answer scale with visual aid**


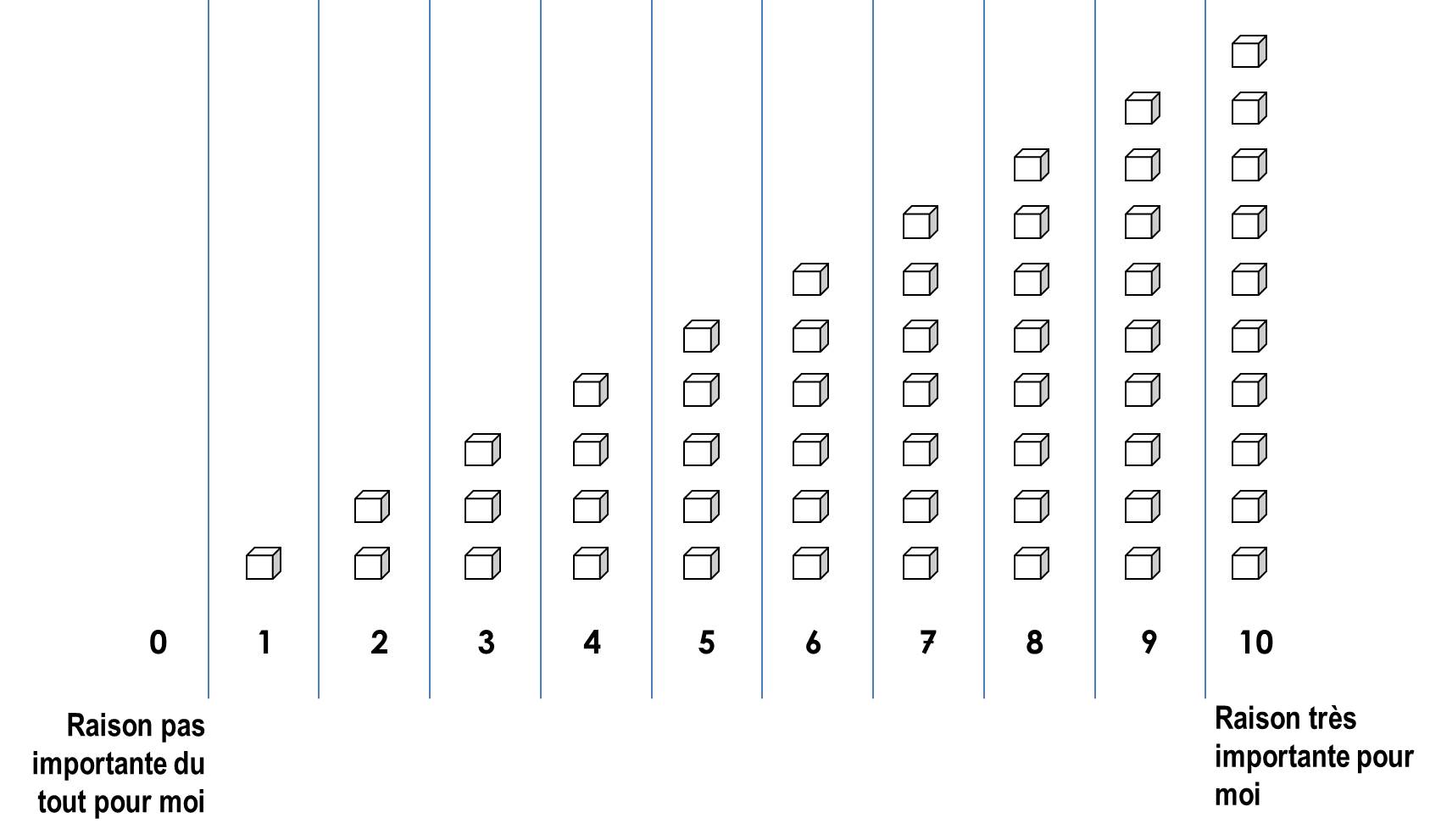


English equivalent of the poles: 0 = “not important to me at all”; 10 = “extremely important to me”

**Full 26-item scale**

Note that the English translation is intended to facilitate understanding for the non-French-speaking readership. It is not tested and validated and might thus not be perfectly equivalent to the French version.

As described in the main paper, this list was reduced to 15 items in the context of the validity analyses. Non-retained items are marked with an asterisk. In the table, items are organized by motivation dimensions. However, they were presented in different order in the actual questionnaire. This order is indicated by the last column in the table.

The following question served as an overarching guiding question:

**Pourquoi êtes-vous motive à travailler ? - Why are you motivated to work?**

| Intrinsic motivation (IM) | Parce que j’aime faire ce que je fais chaque jour au travail.  *Because I enjoy doing what I do at work every day.* | 7 |
| --- | --- | --- |
|  | Parce que mes tâches au travail me plaisent beaucoup.  *Because I enjoy my work tasks.* | 12 |
|  | Parce que le travail que je fais est très intéressant.  *Because the work that I do is very interesting.* | 25 |
|  | *Parce que ce travail me plaît beaucoup.  *Because I very much like doing this job.* | 5 |
|  | *Parce que j'aime faire face aux défis que je rencontre dans mon travail.  *Because I like the challenges I face in my work.* | 9 |
|  | *Parce qu'être en contact avec beaucoup de gens chaque jour me plaît beaucoup.  *Because I enjoy interacting with many people every day.* | 15 |
| Integrated regulation (INTEG) | Parce qu’être un agent de santé est un élément fondamental de ce que je suis.  *Because being a health worker is a fundamental part of who I am.* | 17 |
|  | *Parce que je ne serai pas moi-même si je n'étais pas là pour prendre soin de mes patients.  *Because I wouldn't be me if I wasn't there to care for my patients.* | 23 |
|  | *Parce que mon travail est plus qu'un métier, c'est une vocation.  *Because my work is more than a job, it’s a mission.* | 1 |
|  | *Parce que je ne peux pas m'imaginer être autre chose qu'un agent de santé.  *Because I can't see myself as anything else than a health worker.* | 14 |
| Identified regulation (IDEN) | Parce que mon travail est extrêmement important pour mes patients.  *Because my work is extremely important for my patients.* | 19 |
|  | Parce que je veux changer quelque chose dans la vie des autres.  *Because I want to make a difference in people’s lives.* | 16 |
|  | *Parce que mon travail me permet d'atteindre mes objectifs dans la vie.  *Because my job allows me achieve my goals in life.* | 20 |
|  | *Parce que ce travail correspond très bien à mes valeurs personnelles.  *Because this job fits my personal values very well.* | 4 |
| Introjected regulation (INTRO) | Pour avoir une bonne opinion de moi-même.  *In order to feel good about myself.* | 13 |
|  | Parce que ma réputation dépend de mon travail.  *Because my reputation depends on my work.* | 22 |
|  | * Parce que mon travail me rend fier de moi.  *Because my work makes me feel proud of myself.* | 6 |
|  | * Parce que autrement j'aurais honte de moi.  *Because I would feel ashamed otherwise.* | 2 |
|  | * Parce que m'occuper de mes patients est mon devoir.  *Because it is my duty to care for my patients.* | 10 |
| External regulation (EXT) | A cause de la reconnaissance que je reçois de mes patients et de la communauté.  *Because of the appreciation I receive from my patients and the community.* | 26 |
|  | Pour ne pas laisser tomber mon équipe.  *So I don't let my team down.* | 21 |
|  | Parce que mon responsable direct reconnaît mon travail et m’apprécie.  *Because my supervisor recognizes and appreciates me.* | 11 |
|  | A cause des avantages liés à mon travail.  *Because of the benefits that come with my job.* | 3 |
|  | Pour pouvoir subvenir aux besoins de ma famille.  *In order to be able to provide for my family.* | 8 |
|  | Parce que mon travail me procure la sécurité financière.  *Because of the financial security my job provides me with.* | 24 |
|  | Afin de gagner de l’argent.  *In order to earn money.* | 18 |

1. **To what extent were the design decisions based on the Malawi experience successful in counteracting challenges encountered in Malawi?**

**Did the more specific and direct item phrasing improve respondents’ ability to handle the scale?**

Although we were unable to assess this systematically, our general impression from the field was that health workers in Burkina Faso were able to handle the more specific phrasing of the items of the newly developed scale much better than health workers in Malawi were able to handle the generic items of the WEIMS.

**Did the increased number of categories, the framing exercise, and the hybrid mode of administration succeed in counteracting social desirability and acquiescence biases (as reflected in relatively lower scoring)?**

Unfortunately, even with all the above measures in place, scores were not substantially lower than they had been in Malawi with the 5-point scale, see distribution plots below. It is difficult to say, however, whether the measures in place were simply not effective in counteracting potential biases, or whether nurses in Burkina Faso actually place such high importance on many of the reasons as is implied by their scoring.

What the 11-point scale did achieve was somewhat more variation ‘at the top’ as compared to Malawi, where health workers effectively only had two response options to choose from if they wanted to stay on the positive end of the scale. This is advantageous for a number of pragmatic and statistical reasons. Most importantly, the scale was initially developed to measure change in motivation composition in response to a PBF intervention. If a large proportion of respondents selects the highest score at baseline already, however, and these scores do not accurately reflect true underlying levels of motivation, then measurement of (real) positive change is very difficult (ceiling effect). Further, from a statistical point of view, more variance is generally better as it allows for the detecting of more subtle shifts in motivation composition or for relationships of the motivation dimensions with other variables (assuming that this variance reflects real variance in the underlying construct, not random variation).

However, we cannot rule out the possibility that the large number of response option choices was overwhelming for some respondents, considering that the difference between for instance scores 8 and 9 is not easy and straightforward to conceptualize and choice of category might thus to some extent be arbitrary rather than a perfectly accurate reflection of the true underlying level of (continuous) motivation. It is thus possible that some answers are not perfectly accurate expressions of the importance of the respective reason, but also a reflection of participants’ being overwhelmed with the amount of possible answer choices.

We are currently planning a follow-up study to better understand respondents’ scoring behavior. Our recommendations for researchers using the scale at this point is to try a somewhat lower number of answer categories (7-9).

**
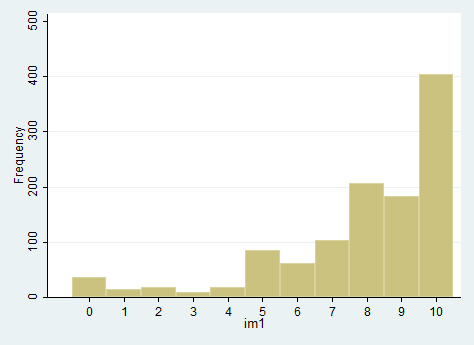

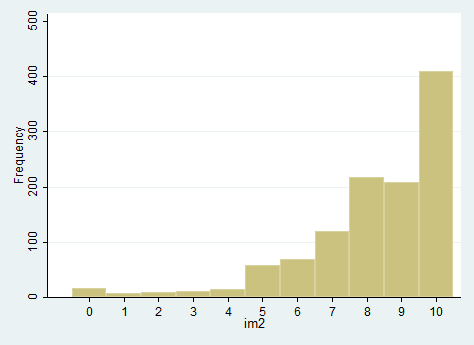

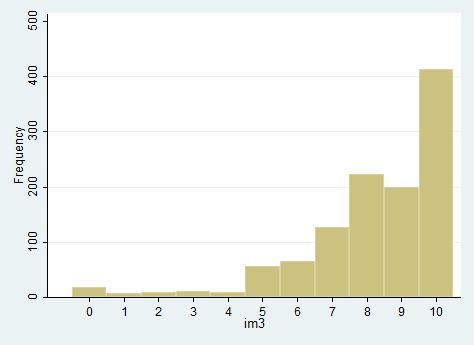

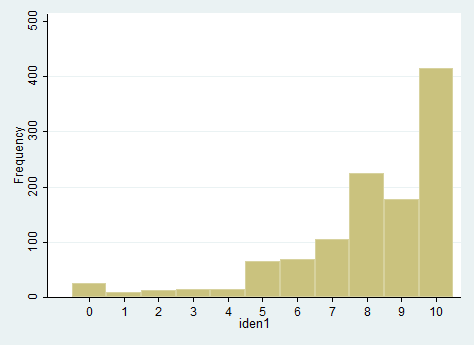

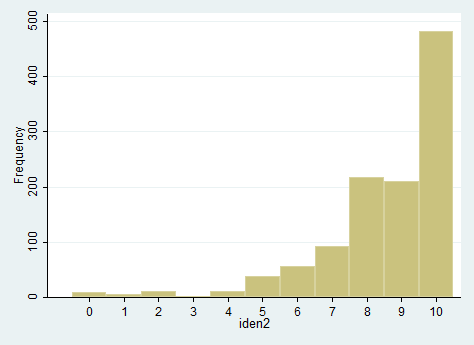

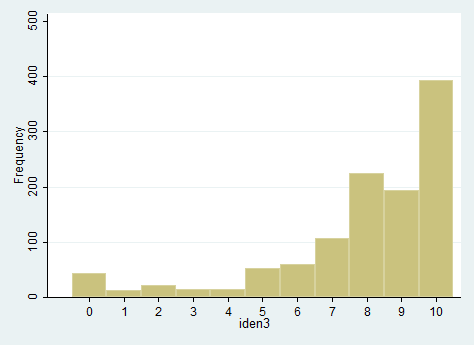

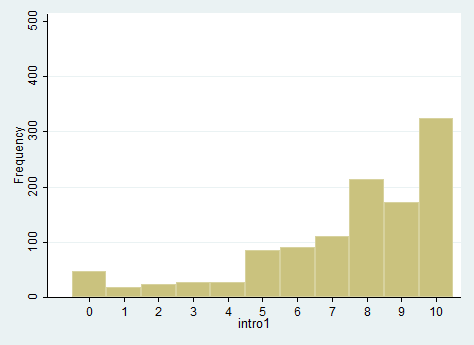

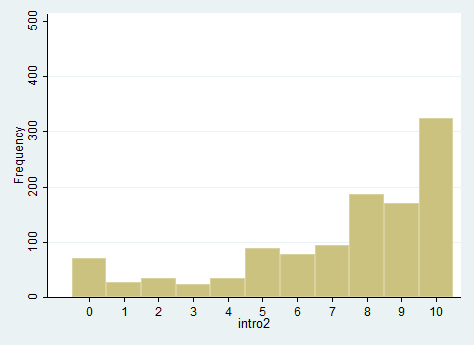

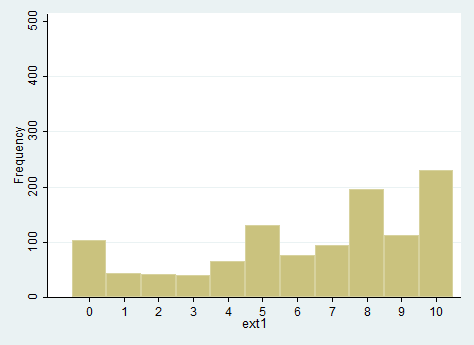

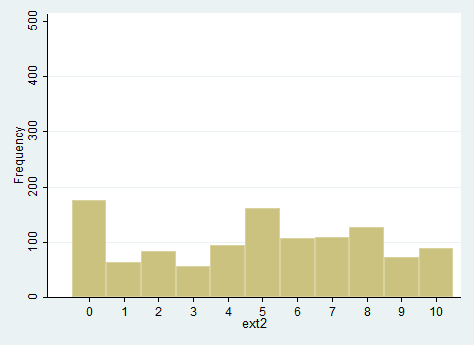

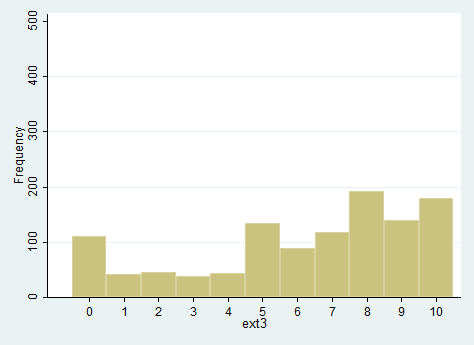

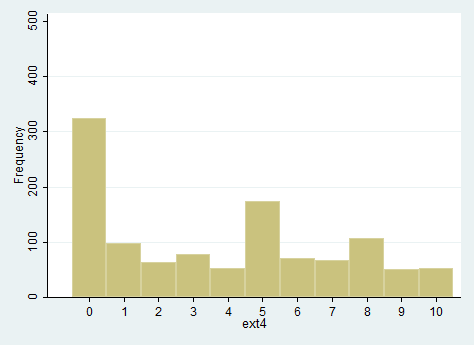

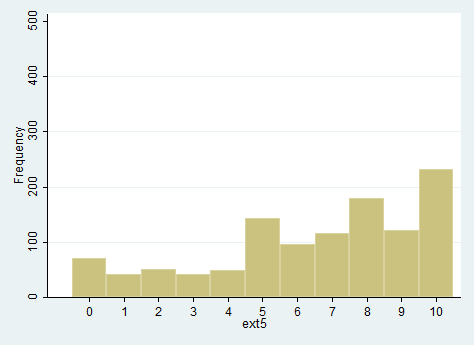

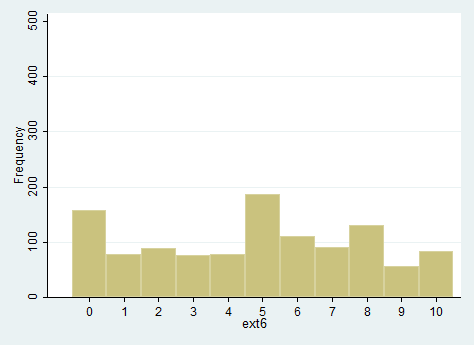

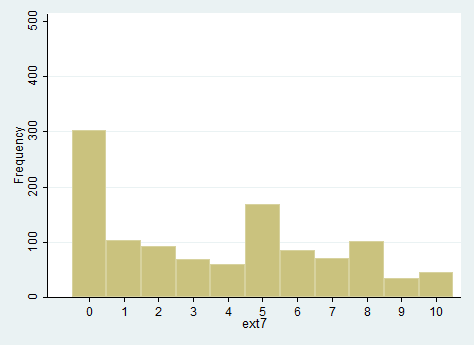
**

1. **References**

[1] Tremblay MA, Blanchard CM, Taylor S, Pelletier LG, Villeneuve M. Work Extrinsic and Intrinsic Motivation Scale. Its value for organizational psychology research. Can J Behav Sci. 2009;41:213-26.

[2] Gagné M, Forest J, Vansteenkiste M, Crevier-Braud L, van den Broeck A, Aspeli AK, et al. The Multidimensional Work Motivation Scale: Validation evidence in seven languages and nine countries. Eur J Work Organ Psychol. 2015;24:178-96.

[3] Ryan RM, Connell JP. Perceived locus of causality and internalization. Examining reasons for acting in two domains. J Pers Soc Psychol. 1989;57:749-61.

[4] Brenner S, Muula AS, Robyn PJ, Bärnighausen T, Sarker M, Mathanga DP, Bossert T, De Allegri M. Design of an impact evaluation using a mixed methods model - an explanatory assessment of the effects of results-based financing mechanisms on maternal healthcare services in Malawi. BMC Health Serv Res. 2014;14:180.

[5] Dieleman M, Cuong PV, Anh LV, Martineau T. Identifying factors for job motivation of rural health workers in North Vietnam. Hum Resour Health. 2003;1:10.

[6] Dieleman M, Toonen J, Touré H, Martineau T. The match between motivation and performance management of health sector workers in Mali. Hum Resour Health. 2006;4:2

[7] Dieleman M, Gerretsen B, van der Wilt GJ. Human resource management interventions to improve health workers’ performance in low and middle income countries. A realist review. Health Res Policy Syst. 2009;7:7.

[8] Henderson LN, Tulloch J. Incentives for retaining and motivating health workers in Pacific and Asian countries. Hum Resour Health. 2008;6:18.

[9] Franco LM, Bennett S, Kanfer R, Stubblebine P. Determinants and consequences of health worker motivation in hospitals in Jordan and Georgia. Soc Sci Med. 2004;58:343-55.

[10] Bennett S, Franco LM, Kanfer R, Stubblebine P. The development of tools to measure the determinants and consequences of health worker motivation in developing countries. Bethesda: Abt. Associates; 2001.

[11] Prytherch H, Leshabari MT, Wiskow C, Aninanya GA, Kakoko DCV, Kagoné M, et al. The challenges of developing an instrument to assess health provider motivation at primary care level in rural Burkina Faso, Ghana and Tanzania. Glob Health Action. 2012;5:1-18.

[12] Mbindyo PM, Blaauw D, Gilson L, English M. Developing a tool to measure health worker motivation in district hospitals in Kenya. Hum Resour Health. 2009;7:40.

[13] Gagné M, Deci EL. Self-determination theory and work motivation. J Organ Behav. 2005;26:331-62.

[14] Paul F. Health worker motivation and the role of performance based finance systems in Africa. A qualitative study on health worker motivation and the Rwandan performance based finance initiative in district hospitals. Master’s thesis. London: London School of Economics; 2009.

[15] Chandler CIR, Chonya S, Mtei F, Reyburn H, Whitty CJM. Motivation, money and respect: a mixed-method study of Tanzanian non-physician clinicians. Soc Sci Med. 2009;68:2078–88.

[16] Peters DH, Chakraborty S, Mahapatra P, Steinhardt L. Job satisfaction and motivation of health workers in public and private sectors: cross-sectional analysis from two Indian states. Hum Resour Health. 2010;8:27.

[17] Goldberg AB, Ron Levey I. Understanding the complex drivers of intrinsic motivation for health workers in Malawi. Health Systems 20/20 project report. Bethesda: Abt Associates; 2012.

[18] Gagné M, Forest J, Gilbert MH, Aubé C, Morin E, Malorni A. The Motivation at Work Scale. Validation evidence in two languages. Educ Psychol Meas. 2010;70:628-46.

1. The Malawian study was funded by the United States Agency for International Development under Translating Research into Action, Cooperative Agreement No. GHS-A-00-09-00015-00. [↑](#footnote-ref-1)
2. We deferred the final item selection to the main validation study due to the small pretest sample size, limiting possibilities for robust psychometric assessment, and the minor changes applied following the pretest [↑](#footnote-ref-2)
3. Staff members of our local research partner for the scale development process, the Centre de Recherche en Santé de Nouna, as well as informal interviews with nurses in the context of the pretest [↑](#footnote-ref-3)
