# Supplementary material for: Measuring health workers’ motivation composition: validation of a scale based on Self-Determination Theory in Burkina Faso
Source: Hum Resour Health. 2017 May 22;15:33. doi: 10.1186/s12960-017-0208-1 (PMC5441099; doi:10.1186/s12960-017-0208-1)
Supplement: Supplementary file 2 — Items eliminated in the analytical process, standardized parameter estimates for Model C, and suggested modification indices for Model C. (DOCX 107 kb) [file 12960_2017_208_MOESM2_ESM.docx]

**Items eliminated in the analytical process**

| **Subscale** | **Item** | **Reasons for elimination** | **Stage of elimination** |
| --- | --- | --- | --- |
| Intrinsic motivation | Parce que ce travail me plaît beaucoup.  *Because I very much like doing this job.* | Very high scoring, very little discrimination between respondents | Initial item analysis |
|  | Parce que j’aime faire face aux défis que je rencontre dans mon travail.  *Because I like the challenges I face in my work.* | Suboptimal phrasing: more specific than other IM items, pertaining only to intrinsic interest in one aspect of the job, while others are more generic | Initial item analysis |
|  | Parce qu’être en contact avec beaucoup de gens chaque jour me plaît beaucoup.  *Because I enjoy interacting with many people every day.* | Suboptimal phrasing: more specific than other IM items, pertaining only to intrinsic interest in one aspect of the job, while others are more generic; high correlations with other items with a ‘social connotation’ | Initial item analysis |
| Integrated/ identified regulation | Parce que je ne serai pas moi-même si je n’étais pas là pour prendre soin de mes patients.  *Because I wouldn't be me if I wasn't there to care for my patients.* | Poor performance in the model | CFA (structural validation) |
|  | Parce que mon travail est plus qu’un métier, c’est une vocation.  *Because my work is more than a job, it’s a mission.* | Very high scoring, very little discrimination between respondents; phrasing suboptimal (suggestive) | Initial item analysis |
|  | Parce que je ne peux pas m’imaginer être autre chose qu’un agent de santé.  *Because I can't see myself as anything else than a health worker.* | Does not cluster with other items in a semantically meaningful way | Initial item analysis |
|  | Parce que mon travail me permet d’atteindre mes objectifs dans la vie.  *Because my job allows me achieve my goals in life.* | Low correlation with subscale items, but high correlation with external-material items indicates that item might be understood in a material/financial sense | Initial item analysis |
|  | Parce que ce travail correspond très bien à mes valeurs personnelles.  *Because this job fits my personal values very well.* | Poor performance in the model | CFA (structural validation) |
| Introjected regulation | Parce que mon travail me rend fier de moi.  *Because my work makes me feel proud of myself.* | Poor performance in the model | CFA (structural validation) |
|  | Parce qu’autrement j’aurais honte de moi-même.  *Because I would feel ashamed otherwise.* | Does not correlate with any other items in the scale | Initial item analysis |
|  | Parce que m’occuper de mes patients est mon devoir.  *Because it is my duty to care for my patients.* | Very high scoring, very little discrimination between respondents; likely understood in terms of job requirements rather than introjected feelings of duty beyond mere job requirements | Initial item analysis |

**Standardized parameter estimates for Model C**

**
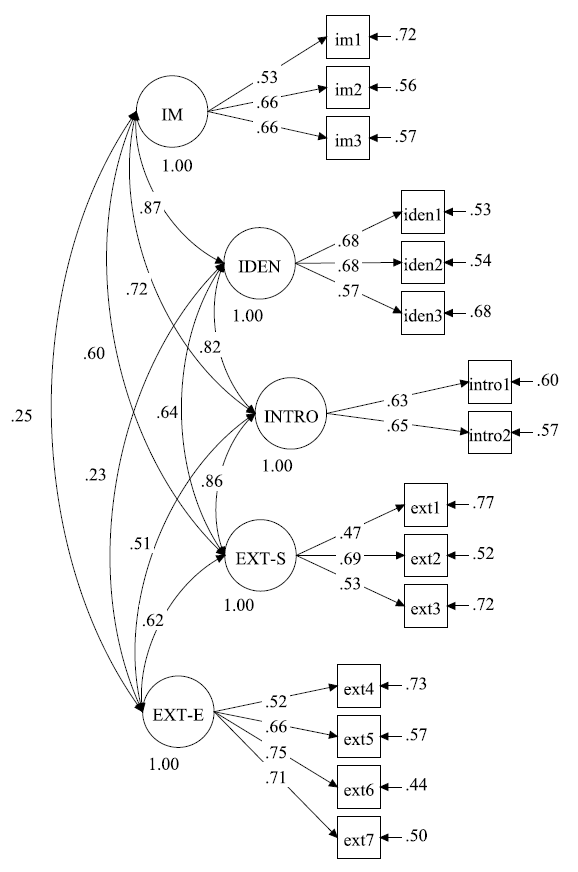
**

Legend: Coefficients attached to arrows pointing at items from the right: residual variances (i.e. non-explained item variance, error terms); coefficients attached to arrows from factors to items: factor loadings; factor variances were fixed to 1 for model identification; coefficients attached to curved arrows represent factor correlations

**Suggested modification indices for Model C**

Modification indices signal by how much model χ2 would decrease (i.e. by how much overall model fit would increase) if the respective path was explicitly modelled and thus freely estimated rather than implicitly set to zero. In model C, χ2 (227 in the above specification) would need to decrease by at least 124 in order to result in a non-significant p(.05)-value.

Non-modelled item – non-target-factor loadings (‘cross-loadings’)

| IDEN1 | on | IM | 3.35 |
| --- | --- | --- | --- |
| IDEN2 | on | IM | 0.14 |
| IDEN3 | on | IM | 2.66 |
| INTRO1 | on | IM | 16.56 |
| INTRO2 | on | IM | 16.55 |
| EXT1 | on | IM | 3.45 |
| EXT2 | on | IM | 4.91 |
| EXT3 | on | IM | 0.64 |
| EXT4 | on | IM | 0.06 |
| EXT5 | on | IM | 1.33 |
| EXT6 | on | IM | 9.29 |
| EXT7 | on | IM | 16.91 |
| IM1 | on | IDEN | 0.08 |
| IM2 | on | IDEN | 1.45 |
| IM3 | on | IDEN | 0.83 |
| INTRO1 | on | IDEN | 2.51 |
| INTRO2 | on | IDEN | 2.51 |
| EXT1 | on | IDEN | 2.22 |
| EXT2 | on | IDEN | 2.56 |
| EXT3 | on | IDEN | 0.19 |
| EXT4 | on | IDEN | 0.00 |
| EXT5 | on | IDEN | 1.86 |
| EXT6 | on | IDEN | 5.37 |
| EXT7 | on | IDEN | 13.73 |
| IM1 | on | INTRO | 1.24 |
| IM2 | on | INTRO | 1.74 |
| IM3 | on | INTRO | 0.11 |
| IDEN1 | on | INTRO | 2.42 |
| IDEN2 | on | INTRO | 0.05 |
| IDEN3 | on | INTRO | 4.23 |
| EXT1 | on | INTRO | 0.63 |
| EXT2 | on | INTRO | 0.03 |
| EXT3 | on | INTRO | 0.32 |
| EXT4 | on | INTRO | 0.11 |
| EXT5 | on | INTRO | 1.52 |
| EXT6 | on | INTRO | 7.04 |
| EXT7 | on | INTRO | 17.31 |
| IM1 | on | EXT_S | 1.83 |
| IM2 | on | EXT_S | 3.29 |
| IM3 | on | EXT_S | 0.44 |
| IDEN1 | on | EXT_S | 2.67 |
| IDEN2 | on | EXT_S | 0.06 |
| IDEN3 | on | EXT_S | 4.81 |
| INTRO1 | on | EXT_S | 0.75 |
| INTRO2 | on | EXT_S | 0.75 |
| EXT4 | on | EXT_S | 0.73 |
| EXT5 | on | EXT_S | 0.09 |
| EXT6 | on | EXT_S | 6.09 |
| EXT7 | on | EXT_S | 11.96 |
| IM1 | on | EXT_E | 1.94 |
| IM2 | on | EXT_E | 3.52 |
| IM3 | on | EXT_E | 0.53 |
| IDEN1 | on | EXT_E | 0.70 |
| IDEN2 | on | EXT_E | 0.10 |
| IDEN3 | on | EXT_E | 1.89 |
| INTRO1 | on | EXT_E | 0.60 |
| INTRO2 | on | EXT_E | 0.60 |
| EXT1 | on | EXT_E | 0.02 |
| EXT2 | on | EXT_E | 0.55 |
| EXT3 | on | EXT_E | 0.51 |

Residual correlations

Residual correlations are correlations of item error terms (i.e. item variance unexplained by the model); substantive reasons are often similar item phrasing and other item similarities beyond the shared latent variable. As for cross-loadings, the numbers signal the potential decrease in χ2 if the residual correlations were explicitly modelled.

| IM2 | with | IM1 | 2.08 |
| --- | --- | --- | --- |
| IM3 | with | IM1 | 5.03 |
| IM3 | with | IM2 | 0.58 |
| IDEN1 | with | IM1 | 0.16 |
| IDEN1 | with | IM2 | 0.91 |
| IDEN1 | with | IM3 | 8.33 |
| IDEN2 | with | IM1 | 0.43 |
| IDEN2 | with | IM2 | 1.37 |
| IDEN2 | with | IM3 | 0.93 |
| IDEN2 | with | IDEN1 | 0.16 |
| IDEN3 | with | IM1 | 0.41 |
| IDEN3 | with | IM2 | 6.63 |
| IDEN3 | with | IM3 | 0.09 |
| IDEN3 | with | IDEN1 | 0.15 |
| IDEN3 | with | IDEN2 | 0.66 |
| INTRO1 | with | IM1 | 0.95 |
| INTRO1 | with | IM2 | 39.51 |
| INTRO1 | with | IM3 | 2.52 |
| INTRO1 | with | IDEN1 | 0.00 |
| INTRO1 | with | IDEN2 | 4.98 |
| INTRO1 | with | IDEN3 | 1.74 |
| INTRO2 | with | IM1 | 1.98 |
| INTRO2 | with | IM2 | 15.11 |
| INTRO2 | with | IM3 | 0.07 |
| INTRO2 | with | IDEN1 | 0.02 |
| INTRO2 | with | IDEN2 | 4.82 |
| INTRO2 | with | IDEN3 | 2.03 |
| EXT1 | with | IM1 | 2.24 |
| EXT1 | with | IM2 | 0.00 |
| EXT1 | with | IM3 | 8.72 |
| EXT1 | with | IDEN1 | 0.00 |
| EXT1 | with | IDEN2 | 3.11 |
| EXT1 | with | IDEN3 | 0.98 |
| EXT1 | with | INTRO1 | 1.37 |
| EXT1 | with | INTRO2 | 0.15 |
| EXT2 | with | IM1 | 0.84 |
| EXT2 | with | IM2 | 9.78 |
| EXT2 | with | IM3 | 0.20 |
| EXT2 | with | IDEN1 | 2.98 |
| EXT2 | with | IDEN2 | 2.63 |
| EXT2 | with | IDEN3 | 10.49 |
| EXT2 | with | INTRO1 | 1.29 |
| EXT2 | with | INTRO2 | 12.94 |
| EXT2 | with | EXT1 | 2.39 |
| EXT3 | with | IM1 | 1.16 |
| EXT3 | with | IM2 | 5.85 |
| EXT3 | with | IM3 | 4.48 |
| EXT3 | with | IDEN1 | 0.01 |
| EXT3 | with | IDEN2 | 0.42 |
| EXT3 | with | IDEN3 | 0.58 |
| EXT3 | with | INTRO1 | 1.42 |
| EXT3 | with | INTRO2 | 9.09 |
| EXT3 | with | EXT1 | 0.65 |
| EXT3 | with | EXT2 | 0.73 |
| EXT4 | with | IM1 | 6.24 |
| EXT4 | with | IM2 | 5.01 |
| EXT4 | with | IM3 | 0.75 |
| EXT4 | with | IDEN1 | 0.61 |
| EXT4 | with | IDEN2 | 0.74 |
| EXT4 | with | IDEN3 | 0.18 |
| EXT4 | with | INTRO1 | 0.01 |
| EXT4 | with | INTRO2 | 0.28 |
| EXT4 | with | EXT1 | 0.08 |
| EXT4 | with | EXT2 | 1.91 |
| EXT4 | with | EXT3 | 1.26 |
| EXT5 | with | IM1 | 2.53 |
| EXT5 | with | IM2 | 0.00 |
| EXT5 | with | IM3 | 1.25 |
| EXT5 | with | IDEN1 | 0.07 |
| EXT5 | with | IDEN2 | 2.90 |
| EXT5 | with | IDEN3 | 1.15 |
| EXT5 | with | INTRO1 | 4.98 |
| EXT5 | with | INTRO2 | 0.00 |
| EXT5 | with | EXT1 | 0.09 |
| EXT5 | with | EXT2 | 11.73 |
| EXT5 | with | EXT3 | 2.19 |
| EXT5 | with | EXT4 | 0.33 |
| EXT6 | with | IM1 | 0.45 |
| EXT6 | with | IM2 | 3.54 |
| EXT6 | with | IM3 | 8.48 |
| EXT6 | with | IDEN1 | 0.50 |
| EXT6 | with | IDEN2 | 1.25 |
| EXT6 | with | IDEN3 | 1.65 |
| EXT6 | with | INTRO1 | 0.22 |
| EXT6 | with | INTRO2 | 9.10 |
| EXT6 | with | EXT1 | 0.98 |
| EXT6 | with | EXT2 | 0.01 |
| EXT6 | with | EXT3 | 1.12 |
| EXT6 | with | EXT4 | 12.09 |
| EXT6 | with | EXT5 | 0.94 |
| EXT7 | with | IM1 | 1.56 |
| EXT7 | with | IM2 | 4.73 |
| EXT7 | with | IM3 | 0.59 |
| EXT7 | with | IDEN1 | 0.00 |
| EXT7 | with | IDEN2 | 0.00 |
| EXT7 | with | IDEN3 | 6.15 |
| EXT7 | with | INTRO1 | 4.30 |
| EXT7 | with | INTRO2 | 6.03 |
| EXT7 | with | EXT1 | 0.06 |
| EXT7 | with | EXT2 | 6.20 |
| EXT7 | with | EXT3 | 2.34 |
| EXT7 | with | EXT4 | 10.94 |
| EXT7 | with | EXT5 | 0.04 |
| EXT7 | with | EXT6 | 0.00 |
